# Innovative Formulation Combining Al, Zr and Si Precursors to Obtain Anticorrosion Hybrid Sol-Gel Coating

**DOI:** 10.3390/molecules23051135

**Published:** 2018-05-10

**Authors:** Clément Genet, Marie-Joëlle Menu, Olivier Gavard, Florence Ansart, Marie Gressier, Robin Montpellaz

**Affiliations:** 1 CIRIMAT, Université de Toulouse, CNRS INPT UPS, UMR 5085, 118 Route de Narbonne, 31062 Toulouse CEDEX 09, France; menu@chimie.ups-tlse.fr (M.-J.M.); ansart@chimie.ups-tlse.fr (F.A.); gressier@chimie.ups-tlse.fr (M.G.); 2 Amphenol Socapex, 948 Promenade de l’Arve–BP 29, 74300 Thyez, France; Olivier.Gavard@amphenol-socapex.fr (O.G.); Robin.Montpellaz@amphenol-socapex.fr (R.M.)

**Keywords:** aluminium alloy 6061, sol-gel process, hybrid coating, corrosion resistance

## Abstract

The aim of our study is to improve the aluminium alloy corrosion resistance with Organic-Inorganic Hybrid (OIH) sol-gel coating. Coatings are obtained from unusual formulation with precursors mixing: glycidoxypropyltrimethoxysilane (GPTMS), zirconium (IV) propoxide (TPOZ) and aluminium tri-sec-butoxide (ASB). This formulation was characterized and compared with sol formulations GPTMS/TPOZ and GPTMS/ASB. In each formulation, a corrosion inhibitor, cerium (III) nitrate hexahydrate, is employed to improve the corrosion performance. Coatings obtained from sol based on GPTMS/TPOZ/ASB have good anti-corrosion performances with Natural Salt Spray (NSS) resistance of 500 h for a thickness lower than 4 µm. Contact angle measurement showed a coating hydrophobic behaviour. To understand these performances, nuclear magnetic resonance (NMR) analyses were performed, results make sol-gel coating condensation evident and are in very good agreement with previous results.

## 1. Introduction

Aluminium alloys are used in the aeronautic industry for their high strength and low density [1,2]. AA6061 alloy is the mostly employed because of its good malleability and its better corrosion resistance than AA2024 due to the presence of Mg and Si additional elements [3]. Nevertheless, aluminium and its alloys are sensitive to corrosion and many mechanisms can affect a material’s durability such as: pitting corrosion, transgranular and intergranular corrosion, and erosion [3].

To improve this corrosion resistance, many techniques are used to form a protective layer on aluminium alloys: chemical conversion layer or anodic oxidation combined with sealing and/or organic layer (painting). However, these techniques employ hexavalent chromium and the use of this chemical substance is prohibited by REACH (Registration, Evaluation, Authorization and Restriction of Chemicals) and European Directive RoHS (Restriction of Hazardous Substances) [4,5]. These directives underline all dangerous chemicals substances for the environment and human health. The different industry sectors have to find and develop new solutions environmentally compliant Cr (VI) free.

These last years, new technologies are developed to improve the corrosion resistance of aluminium alloys like Cr (III) conversion or AO (Anodic Oxidation) [6,7,8]. Among these techniques, the sol-gel route is a very promising process. The Boeing company developed the first commercialized sol-gel with Boegel^®^ technology which is used as a painting adhesion promoter.

The sol-gel process can be used to prepare an eco-friendly hybrid protective coating to prevent corrosion by mixing different organic and inorganic metal-alkoxide precursors (M(OR)_n_ with M = Si, Al, Ti, Zr …). Sol-gel route is based on hydrolysis and condensation reactions of M(OR)_n_, to form a hybrid network [9].

According the nature of the alkoxide precursors, organic-inorganic hybrid coatings can be formed (OIH coating can be developed on many substrates such as steel, Mg based alloys, Zn based alloys, Cu based alloys and Al based alloys) [10,11,12]. In his recent review, R.B Figueira noted that GPTMS and TEOS are the main precursors employed (respectively at 72% and 46% in publications) in sol-gel coating development on aluminium and aluminium alloys [12]. Silicate-epoxy coatings have largely been studied and these precursors form silane based coatings, which have a good barrier effect due to the presence of the Si-O-Si network. Hybrid precursors are used for their effect on mechanical properties and especially for shaping and flexibility [13]. To improve the affinity between sol-gel coating and aluminium alloy, an aluminium alkoxide precursor can be used [14] and for durability and barrier properties, zirconium alkoxide is employed as precursors [15,16,17,18,19].

Furthermore, to improve coating corrosion resistance, corrosion inhibitors can be added to sol preparation. These inhibitors can be organic or inorganic [20]. Since recent years, Ce^3+^ cerium ion is largely studied and this inhibitor has proven its efficiency regarding the anticorrosion property [14,21,22].

Finally, the coating performance will depend on some important parameters such as substrate surface preparation, nature of precursors, pH, hydrolysis degree and curing temperature. The good combination of all these parameters will influence coating performances and will impact coating structuration and corrosion resistance [23].

The aim of our study is to combine several precursors in order to develop a new formulation for those multi-functional organic-inorganic hybrid coatings. The sols were prepared from the following precursors: (3-Glycidyloxypropyl)trimethoxysilane (GPTMS), Aluminium-tri-sec-butoxide (ASB) and Zirconium (IV) propoxide (TPOZ) and coatings are shaped by a dip-coating technique [24]. Three sols are synthesized and characterized: GPTMS/TPOZ (GZ sol), GPTMS/ASB (GA sol) and GPTMS/TPOZ/ASB (GZA sol). Originality comes also from the introduction mode of TPOZ zirconium precursor which is usually pre-hydrolysed before its introduction in the final sol [15,16,17,18,19]. Here, the novelty was to combine directly all precursors during sol preparation. Any study using this combination of three precursors was published to our knowledge. Zheludkevich [21] and O’Sullivan [25] have reported coatings obtained from TPOZ and one organoalkoxysilane GPTMS or MAPTMS respectively, the latter highlighting a NSS test corrosion resistance of about 144 h. Acetic acid was employed as a chelating agent for TPOZ [25,26] and also as an acid catalyst. Cerium nitrate hexahydrate was incorporated to the sol to bring Ce^3+^ ions as corrosion inhibitors. Coatings behaviours were compared by Natural Salt Spray test (NSS) to identify the best formulation giving the higher corrosion resistance. Scanning electron microscopy (SEM) together with Nuclear Magnetic Resonance spectroscopy (NMR) have been undertaken to determine coating structuration and condensation degree versus physico-chemical sol parameters.

## 2. Results and Discussion

As sol-gel coating development is run on industrial substrates, surface characterization of AA6061 substrate is necessary to determine the surface roughness before deposition. The knowledge of this parameter, described in a first part, is important to form an efficient covering barrier coating. In a second part, the sol-gel coatings performances obtained from the GA, GZ and GZA sols are compared and evaluated by NSS test to select the best formulation. Then, the coating thickness is varied and analysed to understand its influence on the corrosion behaviour in order to propose an adequate formulation.

### 2.1. AA6061 Substrate Characterization

**Figure 1:**
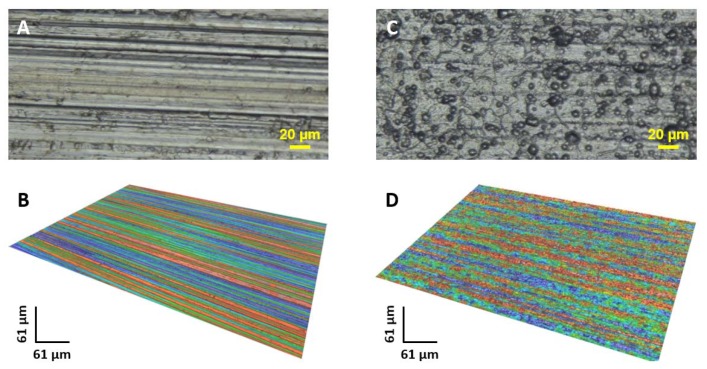
Optical micrographs of surface substrate (**A**) before and (**C**) after surface preparation and respectively (**B**) and (**D**) for the 3D reconstitution.

Before starting the sol-gel coating development it is necessary to determine the surface roughness and the maximum difference between the higher point and lowest point of the substrate surface in order to develop a thick enough coating allowing a long-term protection of the whole surface sample. Surfaces analyses are carried out by optical microscopy and interferometric method and are led before and after the surface preparation (degreasing and etching steps) (Figure 1).

Before surface preparation, rolling ridges are clearly visible (Figure 1A,B). There are no very important surface defects and it is confirmed by interferometric analysis. However, after the surface preparation some cracks can be observed (Figure 1C,D).

The roughness S_a_ value is of 0.50 µm (±0.06) and 0.41 µm (±0.02) before and after surface preparation respectively. The ΔZ_max_ average key parameter is of 1.48 µm (±0.04) and 1.37 (±0.03) before and after surface preparation respectively.

**Figure 2:**
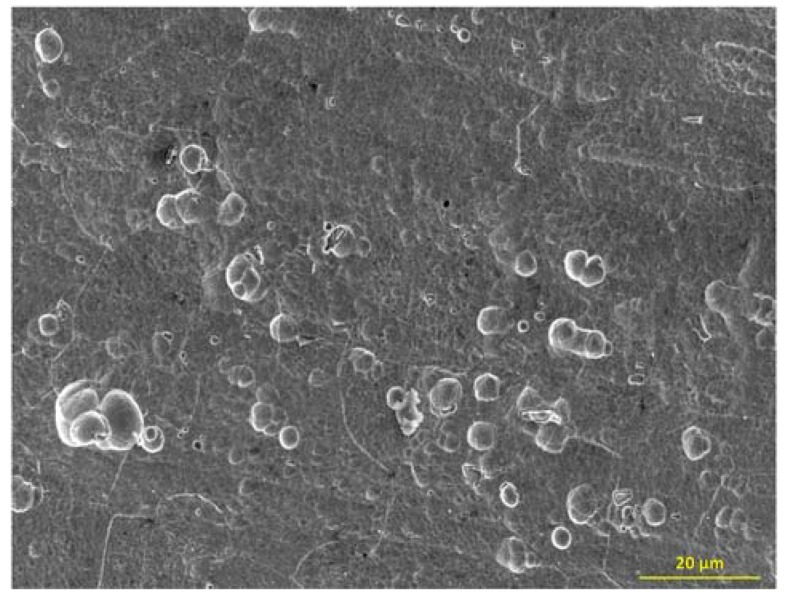
Substrate surface micrograph by scanning electron microscopy-field emission gun (SEM-FEG) after the surface preparation.

The minor difference between the surface roughness before and after the surface preparation can be attributed to a levelling phenomenon of rolling ridges principally during the acid attack. Some holes and/or cracks formations after surface preparation are identified by SEM-FEG and confirm the presence of small crevices and cracks after acid attack (Figure 2).

Following the substrate roughness characterization, sol depositions were realised by dip-coating for the GA, GZ and GZA sols and the corresponding coatings were compared by NSS test resistance.

### 2.2. NSS Tests of Coatings Obtained from GA, GZ and GZA Sols

Many aeronautical specifications require for protective systems of aluminium alloys a corrosion resistance of 500 h NSS exposure. Three coatings are obtained from GZA, GZ and GA sols. Films are deposited by dip-coating with a withdrawal speed corresponding to 400 mm·min^−1^. Curing treatment was the same for all coatings.

**Figure 3:**
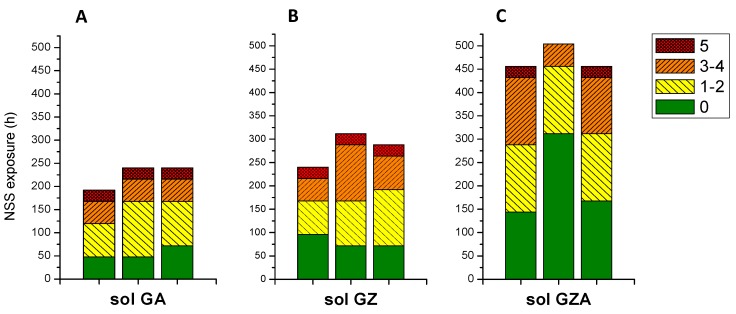
Corrosion resistance to NSS test for coatings obtained from GA (**A**), GZ (**B**) and GZA (**C**) sols.

To discriminate coating performances, three samples of each coating are evaluated by NSS test (Figure 3) and the number of pits (0 to 5) are visualized directly on the graph. On each graph, the plain parts correspond to surface coating without corrosion pitting. The left dashes and the right dashes, correspond respectively to 1 to 2 and 3 to 4 corrosion pits. The small squares correspond to maximum NSS resistance (5 or more corrosion pits). After this, samples were removed of NSS chamber.

This first comparison shows that GZA coating presents a better corrosion resistance than GZ and GA coatings. For one sample of GZA coating (Figure 3C), resistance to NSS is even higher than the recommendation of 500 h. This performance can be attributed to the concomitant presence of aluminium and zirconium in the sol-gel matrix. Furthermore, coating adhesion properties are brought by covalent bonds such as Al-O-Si between the aluminium substrate and the inorganic network as already reported [12,21]. Aluminium element allows increasing adhesion of sol-gel coating on substrate with strong chemical bonds [14]. Furthermore, the zirconium contributes to the barrier effect and improves coating durability [15,16,17,18,19]. This positive aspect can be confirmed by GZ coating, which presents a better corrosion resistance than GA coating (respectively around 300 h against 200 h, (Figure 3B and Figure 3A).

The combination of Zr and Al precursors in the same sol formulation appears to significantly improve the corrosion resistance.

On the basis of these first results, GZA sol was selected and then studied in detail to understand coating structuration before optimizing the coating corrosion resistance.

**Figure 4:**
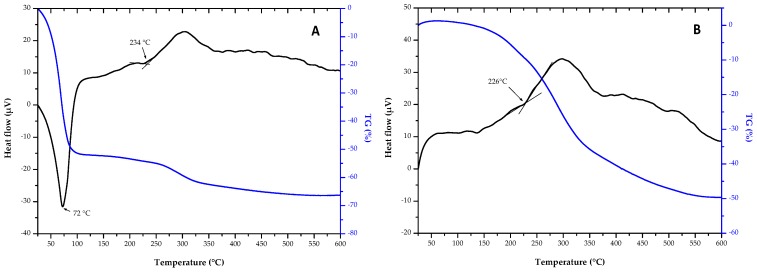
Thermogravimetric-differential (TG-DTA) curves for (**A**) GZA sol and (**B**) GZA xerogel.

Firstly, thermogravimetric-differential (TG-DTA) thermal analyses were realised on GZA sol and xerogel (Figure 4). Tests were performed until 600 °C because of there is no interest to work in higher temperature to avoid silicon and aluminium oxidations.

TG-DTA curve for sol GZA showed endothermic peak until 72 °C with loss mass of 52%. This phenomenon corresponds to solvent evaporation. Then, an exothermic peak was observed starting from 234 °C, with approximatively 10% loss mass, which can be attributed to sol-gel network rearrangement with probably the beginning of the decomposition of the organic part.

On xerogel, an only exothermic peak is identified which corresponds to the same reactions than that observed on TG-DTA curve of the GZA sol. Thermal analysis confirmed that curing treatment applied after dip-coating was well adapted for a good structuration of the hybrid network. Especially, solvents were all removed after curing treatment.

**Figure 5:**
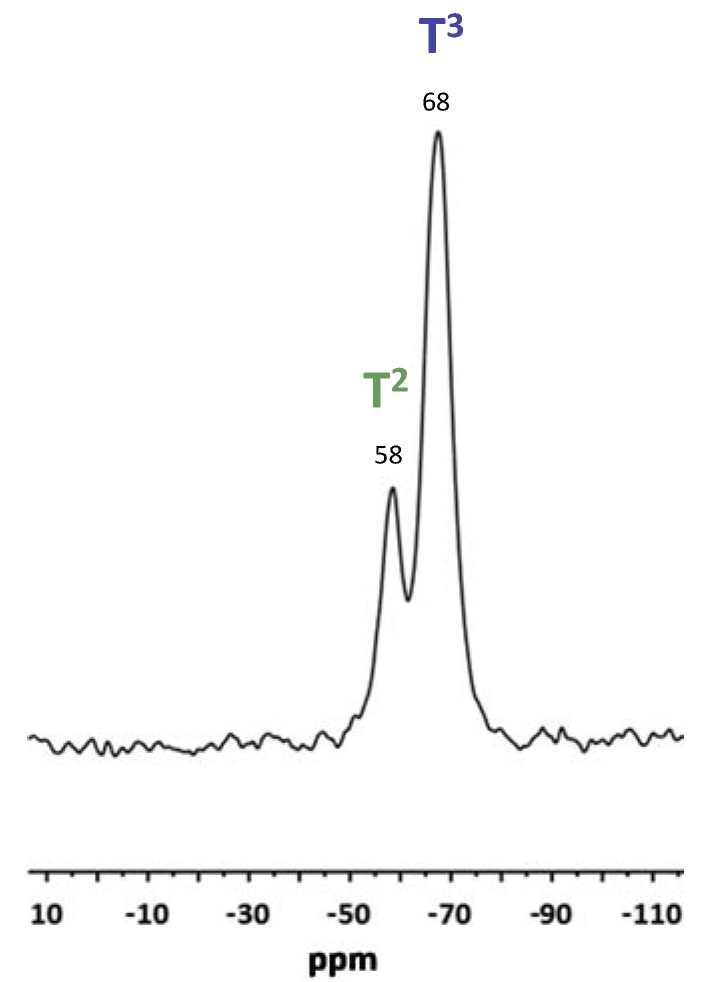
^29^Si Magic angle spinning/nuclear magnetic resonance (MAS/NMR) spectra of GZA xerogel.

The ^29^Si-NMR spectra of GZA xerogel shown in Figure 5 allows to determine the polymerization degree of alkoxysilane functions present in the hybrid network.

**Figure 6:**
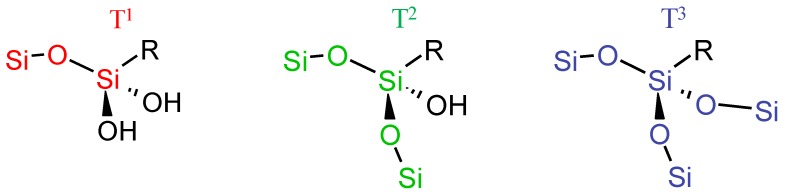
T species silicon atom types, exponent 1, 2 or 3 is related to the presence of one, two or three siloxane Si-O-Si bonds on the silicon atom of the organosilane.

On this spectrum, resonances at −58 and −68 ppm are assigned to the trifunctional unit interconnected through the oxygen atoms to silicon neighbouring, respectively T^2^ (RSi(OSi)_2_(OH) and T^3^ (RSi(OSi)_3_) (Figure 6).

Resonance of trifunctional unit T^1^ RSi(OSi)(OH)_2_ was not observed during acquisition indicating a high condensation ratio of the hybrid matrix. To gain quantitative insight into the relative populations of siloxane environment, ^29^Si MAS NMR spectra were deconvoluted using MestReNova software and the polycondensation degree τ of silicon atom was calculated, in agreement with the equation: τ = (T^1^ + 2T^2^ + 3T^3^)/3 × 100 (Table 1) to give 92% for GZA xerogel [27].

After selected the adequate sol formulation for corrosion resistance and characterized its structuration in order to identify a high polycondensation degree (representative to the barrier property), the coating from sol GZA was optimized to improve corrosion properties.

### 2.3. Optimization of the Coating from Sol GZA

#### 2.3.1. Monolayer Systems

Based on the GZA sol formulation, three coatings were prepared with different withdrawal speeds; 200, 400 and 600 mm·min^−1^. The main aim was to correlate the coating thickness to barrier properties and corrosion resistance.

**Figure 7:**
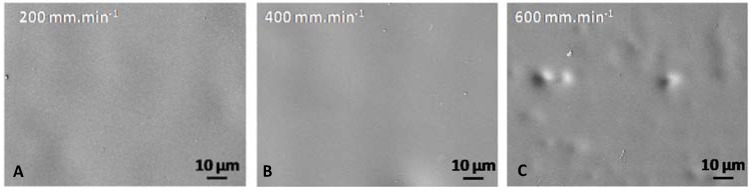
Coating surface micrographs for monolayer systems by scanning electron microscopy (SEM) performed for deposits with withdrawal speed (**A**) 200 mm·min^−1^, (**B**) 400 mm·min^−1^ and (**C**) 600 mm·min^−1^.

In all cases, no defects or cracks are observable on SEM micrographs (Figure 7). Nevertheless, it can be locally observed substrate topography as shown in Figure 7C.

**Figure 8:**
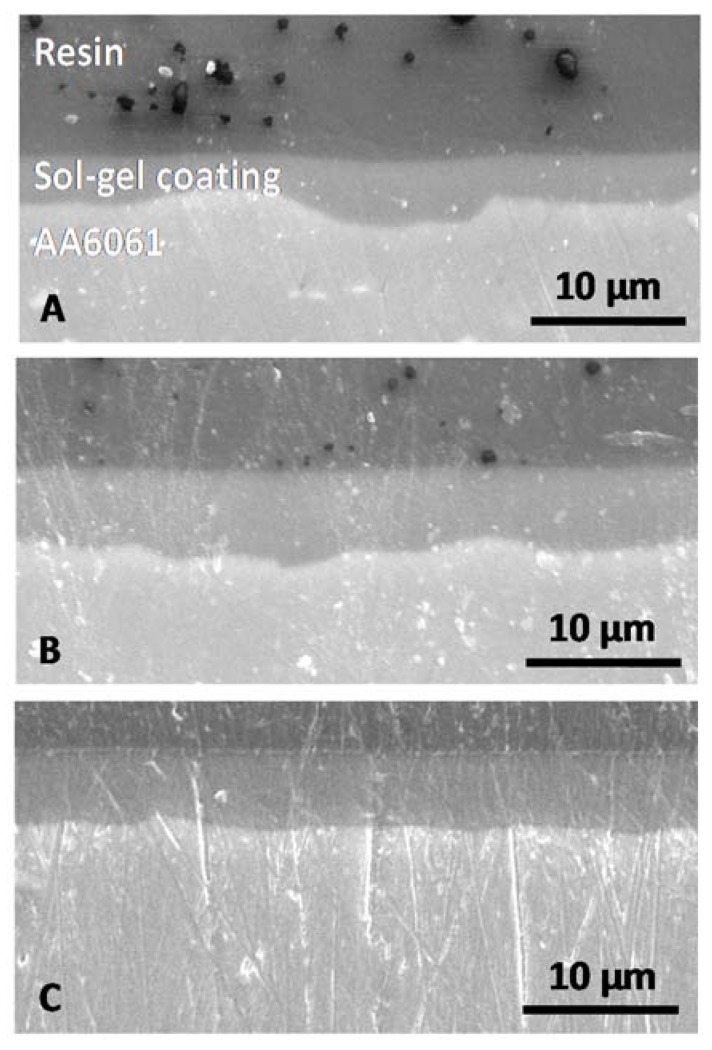
Scanning electron micrographs of cross-section for monolayer systems versus withdrawal speed: (**A**) 200, (**B**) 400 and (**C**) 600 mm·min^−1^.

Thicknesses were then determined by cross section observations (Figure 8) to show their evolution versus the withdrawal speed.

Figure 8 shows that the three coatings are conformal, covering and levelling. The coating thicknesses are determined and reported in Table 2.

Thickness evolves from 2.7 µm to 5.8 µm when withdrawal speed increases. This phenomenon is attributed to the dip-coating technique and is in agreement with the Landau and Levich law [28].

The next step is to evaluate the anti-corrosion behaviour of the three samples by NSS test to estimate the thickness influence on corrosion properties and to prove or not that when thickness increases, barrier properties are improved.

Surprisingly, NSS test shows that the thicker coating (>5 µm) has a corrosion resistance lower than the thinner coatings (≤5 µm). This phenomenon can be attributed to the increase of internal stresses after curing due to a more important deposited matter quantity at the high withdrawal speeds. Curing treatment is probably not adapted in this case because of solvents needed more time to evaporate through the coating. Solvent residues can weaken the coating durability.

To confirm this effect, bilayer systems are developed to obtain coatings thicker than 5 µm.

#### 2.3.2. Bilayer Systems

Withdrawal speeds used were the same as for the monolayer systems, with between each dip-coating deposition a curing treatment of 60 °C during 60 min followed by 120 °C during 120 min.

**Figure 9:**
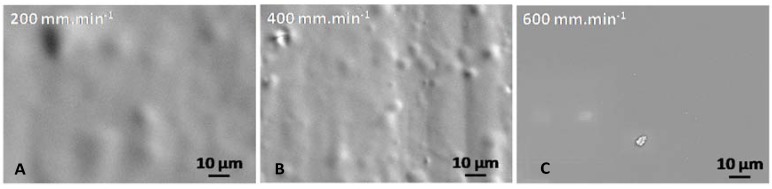
Coating surface micrographs for bilayer systems by SEM performed for deposits with various withdrawal speeds (**A**) 200 mm·min^−1^; (**B**) 400 mm·min^−1^ and (**C**) 600 mm min^−^^1^.

Surface analysis of each coating exhibits that no defect or crack is visible after curing treatment (Figure 9).

Furthermore, on the Figure 9, substrate topography can be identifiable under sol-gel coatings. On the micrograph corresponding to a withdrawal speed of 400 mm·min^−1^, crevices (because of acid attack during surface preparation) and ridges are even observed.

**Figure 10:**
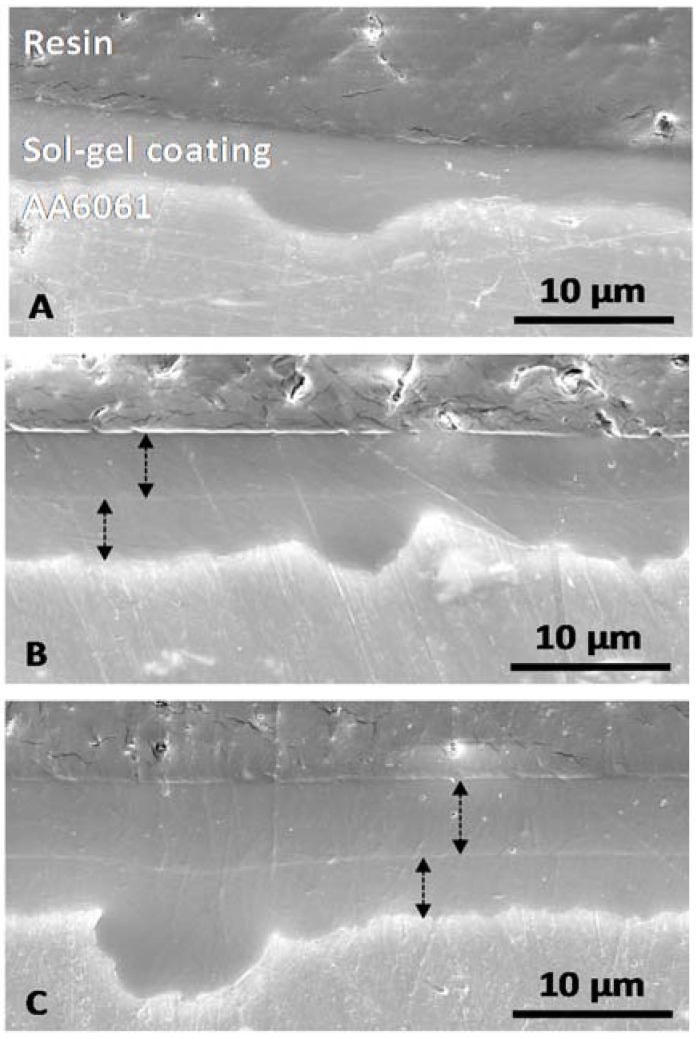
Scanning electron micrographs of cross-section for bilayer systems versus withdrawal speeds: (**A**) 200; (**B**) 400 and (**C**) 600 mm·min^−1^.

Coatings are then analysed in cross-section to evaluate their thickness (Figure 10). When withdrawal speed increases, the coating thickness increases also but compared to monolayer systems, the second deposition contribution is around 2.5 µm or 3.5 µm (Table 3).

For a withdrawal speed 400 mm·min^−1^ and above (Figure 10B,C), the interface between the first and second sol-gel layer is clearly observed. Indeed, the interface is not diffuse while coatings are composed of the same precursors. A good adhesion and affinity, with strong chemical bonds, could have been expected.

As for the monolayer system, coatings obtained from two depositions are conformal, covering and they have a very good levelling effect with a correct ability to fill all surface anfractuosities.

**Figure 11:**
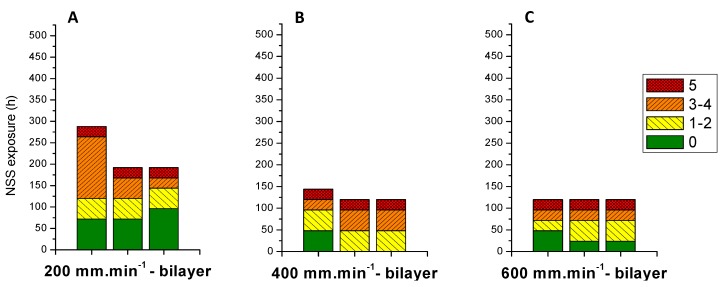
Corrosion resistance to NSS test for bilayer systems obtained from different withdrawal speeds. (**A**): 200 mm·min^−1^; (**B**): 400 mm·min^−1^; (**C**): 600 mm·min^−1^.

Finally, coatings are very continuous without formation of cracks and defects. Bilayer system samples are submitted to NSS test in the same conditions than monolayer systems (Figure 11). The same tendency is observed for bilayer systems, with a decrease of the corrosion resistance when layer thickness increases. The interpretation previously proposed for high thicknesses in monolayer system is confirmed. Furthermore, corrosion resistances after NSS test for bilayer systems are very low compared to monolayer systems (monolayer at 400 mm·min^−1^ average resistance around 450 h versus 144 h to bilayer system at the same withdrawal speed of 400 mm·min^−1^).

To complete NSS test results, contact angle measurements are performed on monolayer and bilayer systems. Contact angles are around 90° for monolayer systems which present rather a hydrophobic behaviour. For bilayer systems, contact angles around 75° showing a slight hydrophilic tendency. These measurements are in good correlation with NSS test, with a risk of electrolyte penetration for bilayer systems.

To explain the differences between these two systems, two interpretations can be suggested:

- A weakening can be generated because of the interface due to the double deposition.
- A critical thickness seems to appear without anti-corrosion properties degradation.

#### 2.3.3. Comparison between Mono and Bilayer System at the Same Thickness

**Figure 12:**
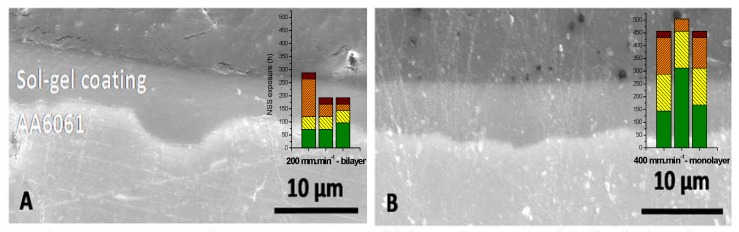
Comparison between (**A**) bilayer and (**B**) monolayer system (similar thickness).

To be free of the thickness influence and to confirm the hypothesis of weakening at the interface between the two sol-gel layers, bi and monolayer systems were compared at the same thickness. Bilayer system was realised at 200 mm·min^−1^ and monolayer at 400 mm·min^−1^ (Figure 12A,B).

The NSS test showed that corrosion resistance for bilayer system was around 200 h versus 450 h for monolayer system for the same coating thickness (respectively 5.3 µm and 5.0 µm).

Therefore, for the same thickness, there is a really important corrosion behaviour difference between mono and bilayer systems. This can prove the weakening of bilayer system and especially at the interface between the two depositions.

**Figure 13:**
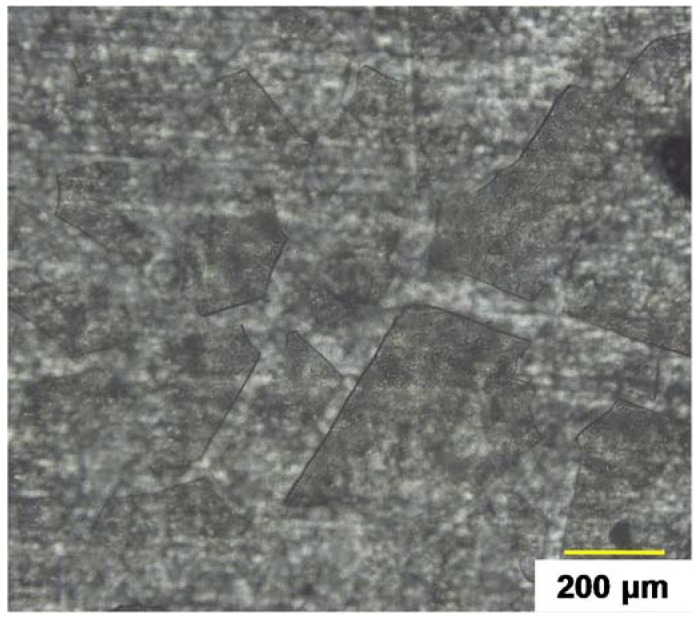
Surface observation for bilayer system at withdrawal speed of 600 mm min^−^^1^.

To make in evidence the interface weakening, optical microscopy was performed after 144 h NSS exposition for bilayer system at withdrawal of 600 mm·min^−1^ (Figure 13).

This observation showed cracking and delamination which are representative to a not sufficient adhesion between two sol-gel layers. The first deposition, in contact with aluminium substrate, endures two curing treatments compared to second sol-gel deposition. This difference modifies the behaviour of the first deposit and formed a layer with a higher density than the top-layer. This can explain the not diffuse interface without chemical bonds to consolidate global sol-gel coating.

Finally, as coatings density are not equivalent, adhesion coefficient is also different which confirms adhesion problem between: (i) substrate AA6061/first sol-gel coating and (ii) the two sol-gel layers.

To put in evidence a weakening for bilayer systems, a study to determine a critical thickness for monolayer systems was then performed.

#### 2.3.4. Critical Thickness Identification

Aluminium alloy 6061 substrates are covered by GZA sol at withdrawal speeds comprised between 200 and 400 mm·min^−1^. For 300 mm·min^−1^, the thickness coating is of 3.5 µm and of 4 µm for 350 mm·min^−1^.

**Figure 14:**
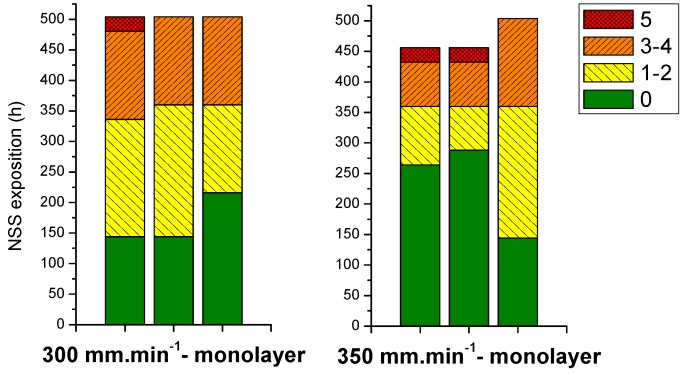
Corrosion resistance to NSS test for coatings obtained from different withdrawal speeds: 300 and 350 mm·min^−1^.

NSS tests showed a corrosion resistance of 450 h for all samples (Figure 14). It is in accordance with the results for monolayer system with a corrosion resistance of 500 h for a thickness under or equal to 5 µm. With these last tests, hybrid sol-gel coatings for good corrosion resistance can be refined to 5 µm at 4 µm.

Finally, critical thickness is established for optimum corrosion resistance with a thickness coating of between 2.5 µm and 4 µm.

**Figure 15:**
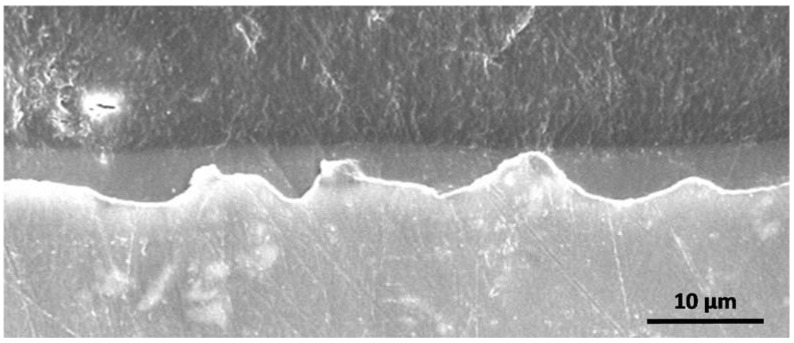
SEM micrograph for coating obtained at withdrawal speed 300 mm·min^−1^.

Now, to explain the fact that a little number of samples does not reach a corrosion resistance of 500 h for thickness in the range [2.5; 4 µm], morphological characterizations are carried out (Figure 15).

In fact, depending on local roughness of the substrate, some parts have a very low thickness sol-gel coating and substrate is not completely covered. These defects can reduce samples corrosion resistance with the apparition of sensitive areas towards pitting corrosion. These results are in accordance with substrate characterization performed and presented in Section 2.1, where substrate roughness is an important parameter to take into account before developing a barrier coating in order to improve corrosion resistance. 

## 3. Experimental

### 3.1. Materials, Sol Composition and Sol-Gel Synthesis

The composition of the AA6061 aluminium alloy is detailed in Table 4.

Samples dimensions are: L = 80 mm, l = 40 mm and e = 1 mm and each sample surface was prepared by two degreasing baths, one acid based on Bonderite AL-85 (Henkel, Boulogne Billancourt, France) and one basic based on Bonderite ETCH-1 (Henkel) and Bonderite T60 (Henkel) and three etching baths composed of nitric acid, sulphuric acid and ammonium hydrogen difluoride. Then, samples were dried under nitrogen.

**Figure 16:**
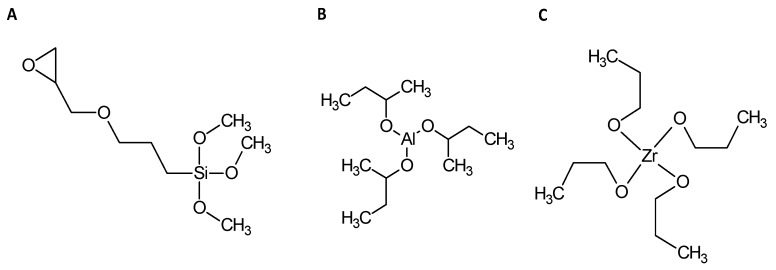
Developed formulas of precursors (**A**) GPTMS, (**B**) ASB and (**C**) TPOZ.

**Figure 17:**
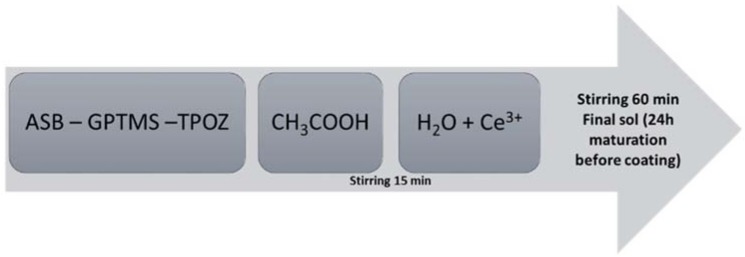
Formulation process for sol preparation.

Sols were prepared from the following precursors: (3-Glycidyloxypropyl)trimethoxysilane (GPTMS, ≥98%, Sigma Aldrich, Lyon, France), Aluminium-tri-sec-butoxide (ASB, 97%, Sigma Aldrich) and Zirconium(IV) propoxide (TPOZ, 70 wt % in 1-propanol, Sigma Aldrich) (Figure 16). Depending on which sol was prepared (GZA, GZ or GA; composition in Table 5), precursors were mixing together with glacial acetic acid (VWR Chemicals, Fontenay-sous-Bois, France). Preparation was stirring 15 min and distilled water with cerium nitrate hexahydrate (Ce(NO_3_)_3_ 6H_2_O, Sigma Aldrich) were added to precursors mixing. Final composition was stirring 60 min and sol was leaving at maturation during 24 h at room temperature (Figure 17).

Sol-gel coatings were shaped after dip-coating process. Withdrawal speed was varied in the range of 200 mm·min^−1^ to 600 mm·min^−1^. Then after deposition, samples were dried at 60 °C during 60 min and curing at 120 °C during 120 min.

### 3.2. Characterization Techniques

#### 3.2.1. Natural Salt Spray Test (NSS)

This technique allows to evaluate anticorrosion coating performance. It is an enclosure chamber and a neutral artificial atmospheric corrosion is reproduced using saline solution at 5 wt % (NaCl in H_2_O). The equipment used is Ascott S12IS model and it was configured according to NF-EN-ISO-9227 and ASTM-B117 (Standard Practice for Operating Salt Spray (Fog) Apparatus).

For each NSS test, three samples were prepared and tested for a good reproducibility. Following the recommendation of the norm, samples are observed every day to have a regular follow-up of their durability. Samples were removed when coated surface presents a minimum of 5 corrosion pits.

#### 3.2.2. Morphological and Surface Characterizations

Coatings microstructure and morphology were analysed by Scanning Electron Microscopy JEOL JSM-6510L (Toulouse, France) and SEM-FEG FEI Quanta 250. (Toulouse, France) Electron field was 20 keV with a distance of 10 mm. Measurements associated with thickness coating were determined with an average minimum of six measurements.

For optical characterisation, Optical Microscope 3D Keyence (Toulouse, France) was used.

Then, for surface characterization, an Interferometric Microscope Sensofar S-Neox (Toulouse, France) was employed. This technique was used to the determination of substrate AA6061 roughness with vertical resolution of 1 nm in interferometric method.

#### 3.2.3. Hydrophobicity by Angle Contact Measurement

Equipment used to the angle contact measurement was a Digidrop Fast 60 goniometer (GBX Scientific Instruments, Toulouse, France). It is an optical technique to determine angle between water drop and surface sample. Micro-syringe allowed to deposit water drop with calibrated volume (4 µL) and drop profile was photographed after 120 ms by Windrop software.

Inscribed triangle was drawn on drop and θ angle was determined by Windrop software. If 0 < θ < 90°, coating surface is qualified of hydrophilic and if 90 > θ > 180° it’s qualified of hydrophobic.

#### 3.2.4. Thermal Analyses (TG-DTA)

Thermals analyses were realised with a Setaram TG-DTA 92 equipment (Toulouse, France). Acquisition was performed under air to 25 °C to 600 °C (heating by step of 5 °C·min^−1^ and cooling by step 10 °C·min^−1^). DTA technique allowed to determine released or absorbed heat when the sample is subjected to physical or chemical transformations. TG technique is used to follow sample mass variation during the heating cycle.

#### 3.2.5. Nuclear Magnetic Resonance (NMR)

Solid state Magic Angle Spinning Nuclear Magnetic Resonance (MAS-NMR) spectra are obtained for ^29^Si by Bruker Avance lIl 400 (9.4T) spectrometer (Toulouse, France). Chemical shift references are tetramethylsilane (TMS) and 4 mm zirconia rotor was used. Rotation speed around magic angle (MAS) was adjusted between 8 kHz and 11 kHz and experiments were realised at room temperature (≈21 °C).

## 4. Conclusions

An original sol-gel formulation has been developed here combining GPTMS (Si), TPOZ (Zr) and ASB (Al) as chemical precursors. Corresponding coatings on AA6061 exhibit a very good corrosion resistance, higher than the required 500 h duration. This performance can be attributed to the formation of an aluminosilicate matrix reinforced by zirconium for the highly condensed inorganic part. The cerium precursor also plays a key role as corrosion inhibitor.

GPTMS content is sufficient to bring cohesion enough and flexibility necessary to obtain crack-free and dense hybrid sol-gel coatings conferring a good barrier property towards corrosive environment.

Corrosion resistance was proved to be efficient for coating thickness in the range from 2.5 µm to 4 µm. Nevertheless, corrosion performance will depend on the substrate roughness and beyond these thickness values, the resistance can be deteriorated especially for bilayer system with weakening interface.

Such very promising performances in the case of so thin coatings have now to be confirmed on aluminium alloy 6061 complex shape parts using the same sol-gel formulation.

Moreover, in the future, this coating can be involved in the development of further multifunctional systems as primer layers.

## Figures and Tables

**Table 1:** T species silicon atom type (%) and condensation ratio of GZA sol-gel network.

Ratio	T^1^	T^2^	T^3^	τ (%)
GZA	0	0.23	0.77	92.3

**Table 2:** Coating thickness for each withdrawal speed-monolayer systems.

Withdrawal Speed (mm·min^−1^)	200	400	600
Thickness (µm)	2.7	5.0	5.8

**Table 3:** Coating thickness for each withdrawal speed-bilayer systems.

Withdrawal Speed (mm·min^−1^)	200	400	600
Thickness (µm)	5.3	8.2	9.5

**Table 4:** Chemical composition of aluminium alloy 6061.

Element	Al	Mg	Si	Fe	Cu	Zn	Mn	Cr	Ti
Wt %	balance	0.8–1.2	0.4–0.8	0.7	0.15–0.4	0.25	0.15	0.04–0.35	0.15

**Table 5:** Sol formulations in molar ratio.

Sol Reference	Si/Al	Si/Zr	CH_3_COOH/Si	CH_3_COOH/Zr	H
GZ	/	2	/	4	4
GA	4	/	1	/	4
GZA	4	2	/	4	4.35
